# Aucubin Promotes Osteogenic Differentiation and Facilitates Bone Formation through the lncRNA-H19 Driven Wnt/*β*-Catenin Signaling Regulatory Axis

**DOI:** 10.1155/2024/5388064

**Published:** 2024-04-09

**Authors:** Yong-xin Mai, Zhi-peng Li, Feng-xiang Pang, Shu-ting Zhou, Nan Li, Yu-yan Wang, Jin-fang Zhang

**Affiliations:** ^1^Cancer Center, Shenzhen Hospital (Futian) of Guangzhou University of Chinese Medicine, Shenzhen 518000, Guangdong, China; ^2^Lingnan Medical Research Center, Guangzhou University of Chinese Medicine, Guangzhou 510405, China; ^3^Department of Rehabilitation, The Sixth Affiliated Hospital, Sun Yat-Sen University, Guangzhou 510655, China; ^4^Department of Traditional Chinese Medicine, The Third Affiliated Hospital, Sun Yat-Sen University, Guangzhou 510630, China

## Abstract

**Objectives:**

Traditional Chinese medicine *Cortex Eucommiae* has been used to treat bone fracture for hundreds of years, which exerts a significant improvement in fracture healing. Aucubin, a derivative isolated from *Cortex Eucommiae*, has been demonstrated to possess anti-inflammatory, immunoregulatory, and antioxidative potential. In the present study, our aim was to explore its function in bone regeneration and elucidate the underlying mechanism.

**Materials and Methods:**

The effects of Aucubin on osteoblast and osteoclast were examined in mouse bone marrow-derived mesenchymal stem cells (BM-MSCs) and RAW 264.7 cells, respectively. Moreover, the lncRNA H19 and Wnt/*β*-catenin signaling were detected by qPCR examination, western blotting, and luciferase activity assays. Using the femur fracture mice model, the *in vivo* effect of Aucubin on bone formation was monitored by X-ray, micro-CT, histomorphometry, and immunohistochemistry staining.

**Results:**

In the present study, Aucubin was found to significantly promote osteogenic differentiation *in vitro* and stimulated bone formation *in vivo*. Regarding to the underlying mechanism, H19 was found to be obviously upregulated by Aucubin in MSCs and thus induced the activation of Wnt/*β*-catenin signaling. Moreover, H19 knockdown partially reversed the Aucubin-induced osteogenic differentiation and successfully suppressed the activation of Wnt/*β*-catenin signaling. We therefore suggested that Aucubin induced the activation of Wnt/*β*-catenin signaling through promoting H19 expression.

**Conclusion:**

Our results demonstrated that Aucubin promoted osteogenesis *in vitro* and facilitated fracture healing *in vivo* through the H19-Wnt/*β*-catenin regulatory axis.

## 1. Introduction

Fracture is a common injury in people's daily life. Normally, long bone fracture needs 2–3 months to recover. Unfortunately, about 5%–10% of patients suffered from fracture delayed healing or nonunion [1]. Its burden is substantial with the loss of function and the decline in the living quality of patients as well as social financial expenditure [2–4]. Therefore, it is of great clinical significance to accelerate fracture healing. Mesenchymal stem cells (MSCs) have been considered as a potential candidate to improve bone formation, and the capacity of osteogenic differentiation is crucial for the bone repair. Hence, discovering strategies to enhance the osteogenesis of MSCs represents a promising avenue for treating fractures. Traditional Chinese medicine *Cortex Eucommiae* has been applied in orthopedics for hundreds of years [5], and it has been reported to ease osteoarthritic knee pain and ameliorate osteoporosis [6, 7]. Aucubin, as a kind of iridoid glycoside, is one of the main active ingredients of *Eucommia* [8]. Several studies have demonstrated that Aucubin exhibits anti-inflammatory, antiosteoporosis, hepatoprotective, and antioxidant activities [8, 9]. Particularly, Aucubin was reported to promote osteogenesis and prevent osteoblast apoptosis [10–12]. However, the detailed mechanism is not fully understood.

Long noncoding RNAs (lncRNAs) are a kind of noncoding transcript with more than 200 nucleotide length [13]. They have been proved to be involved in various biological activities and diseases [14]. A wide variety of lncRNAs has been reported to participate in bone regeneration and bone formation [15, 16]. For instance, lncRNAs MALAT1, TUG1, and HOTAIR were reported to promote osteogenesis and bone formation [17–19]. As one of the earliest discovered lncRNAs, H19 plays crucial roles in various physiological and pathological activities [20–22]. Our previous study found that H19 promoted osteoblast differentiation through activating Wnt/*β*-catenin signaling [23]. We, therefore, wondered whether this H19-driven Wnt/*β*-catenin signaling regulatory axis was involved in the Aucubin-mediated bone formation.

In the present study, our results demonstrated that Aucubin significantly promoted osteogenic differentiation *in vitro*, stimulated new bone formation, and accelerated fracture healing *in vivo*. Further studies discovered that H19 was upregulated by Aucubin, and thus induced the activation of Wnt/*β*-catenin signaling. H19 knockdown rescued the Aucubin-promoted effects on osteogenesis and partially reversed the activation of Wnt/*β*-catenin signaling. Taken together, our results indicated that Aucubin promoted osteogenesis and stimulated bone formation through the H19-mediated Wnt/*β*-catenin signaling regulatory axis, suggesting that Aucubin may be developed as a promising candidate for bone fracture.

## 2. Materials and Methods

### 2.1. Cell Culture

Fresh bone marrow-derived mesenchymal stem cells (BM-MSCs) were isolated from 4-week-old C57BL/6 mice according to previous publication [24]. Briefly, bone marrow was flushed out with a syringe and needle, and cultured in Dulbecco's modified Eagle medium (DMEM) supplemented with 10% FBS and 1% penicillin/streptomycin. The cells were incubated at 37°C with 5% CO_2_ for 7–10 days until nonadherent cells were removed by changing the medium. RAW264.7 were cultured in DMEM (Gibco, Carlsbad, CA) supplemented with 10% fetal bovine serum (Gibco, Carlsbad, CA), 100 U/mL penicillin, and 100 *µ*g/mL streptomycin (Gibco, Carlsbad, CA) at 37°C with a humidity of 5% CO_2_. Aucubin was purchased from Yuanye Bio-Technology (Shanghai, China), dissolved in DMSO, and stored at −20°C for usage.

### 2.2. Cell Viability Assay

Cell viability was examined using the commercially available CCK-8 kit (Beyotime, Shanghai, China). Aucubin with the concentration of 0, 2.5, 5, 10, and 20 *μ*M, or 0, 10, 20, 40, 80, and 160 *μ*M was added into BM-MSCs or RAW264.7, respectively. Then, they were incubated for 24, 48, and 72 hr, and the CCK-8 solution (10 *μ*l/well) was added to each well for 4 hr incubation. The absorbance was measured at 450 nm using the spectrometer (Multiskan GO, Thermo Fisher Scientific, Finland). All the experiments were performed in triplicates and repeated three times.

### 2.3. Osteoblast and Osteoclast Differentiation

The osteogenic differentiation was induced by the classical osteogenic inducers including 10 nM water-soluble dexamethasone (SIGMA, D4902), 50 mM 2-phospho-L-ascorbic acid trisodium salt (SIGMA, 49752) and 10 mM *β*-Glycerophosphate disodium salt hydrate (Macklin, Shanghai, China, cat c12572718). The osteoclast differentiation was induced by 50 ng/mL RANKL.

### 2.4. Alkaline Phosphatase (ALP) Activity Assay and Alizarin Red Staining

BM-MSCs were seeded in 12-well plate and induced to osteoblast differentiation when cells reached 80% confluence. On day 7, the qualitative and quantitative measurements of ALP were conducted using the BCIP/NBT Alkaline phosphatase Color Development Kit (Beyotime, Shanghai, China, cat:c0042) and alkaline phosphatase assay kit (Beyotime, Shanghai, China). On day 21, the cells were washed with PBS and fixed with 75% ethanol for 15 min, then stained with 2% alizarin red S staining solution (pH 4.2, Leagene, Beijing, China) for 30 min. The images were taken by UMAX Powerlook 1120xl (Amersham biosciences, Canon, Japan) and Olympus microscope (Olympus cellsens standard1.18, Olympus, Japan). The stained calcified nodules were dissolved with 10% hexadecylpyridinium chloride monohydrate (Sigma–Aldrich) at room temperature and the absorbance was detected at the wavelength of 562 nm using the spectrometer (Multiskan GO, Thermo Fisher Scientific, Finland).

### 2.5. TRAP and F-Actin Staining

RAW 264.7 cells were seeded at the density of 1,000 cells per well into 96-well plates and added 50 ng/mL RANKL to induce the osteoclast differentiation. Aucubin with various concentrations were added, and the TRAP staining kit (Cosmo bio company, Japan, cat: AK04F) was performed according to the manufacturer's instructions. The images were taken by Olympus microscope (Olympus cellSens standard1.18, Olympus, Japan) and TRAP positive multinucleated cells with three or more nuclei were regarded as Osteoclast-like cells. As for the F-actin staining, the cells were fixed with 4% paraformaldehyde (Biosharp, LOT 1810898) and washed with PBS three times. Then, cells were treated with permeabilized with 0.1% Triton X-100 for 5 min and stained with Rhodamine-Phalloidin (Biorigin, Beijing, BN10063) for 20 min. Subsequently, cell nuclei were stained with DAPI for 5 min and then mounted in antifluorescence quenching agent (Beyotime, Shanghai, China). The images were taken by Olympus microscope (Olympus cellsens standard1.18, Olympus, Japan).

### 2.6. Reverse Transcription Quantitative PCR (RT-qPCR)

Total RNA was extracted from BM-MSCs by TRIzol reagent (Life Invitrogen, America). The cDNA was reversely transcribed from RNA samples by PrimeScriptTM RT Reagent Kit (TaKaRa, Japan). Quantitative PCR reactions were set up in triplicates and performed on a VeritiTM 96-Well Thermal Cycler (Thermo Fisher Scientific, Singapore) using TB GreenTM Premix Ex TaqTM Ⅱ (Tli RNaseH Plus) (TAKARA Bio, Inc.) according to the manufacturer's protocol. The relative expression for each target gene was calculated using the comparative 2^*−ΔΔCT*^ method and normalized to the corresponding *β*-actin values. The primers used in this examination were designed by Sangon Biotech (Shanghai, China) and are listed in Table 1.

### 2.7. Luciferase Activity Assay

BM-MSCs were seeded in 12-well plate and transfected with TOPFlash reporter and pRL-TK vector using the Lipofectamine 3000 Transfection Kit (Invitrogen, North America, LOT2067544). After 12 hr, 5 and 10 *μ*M Aucubin were added into the cells and incubated for 48 hr. The luciferase activities were examined with Dual-Luciferase Reporter Assay System (Promega, USA, lot0000462294).

### 2.8. Western Blotting

The treated cells were harvested and lysed with RIPA lysis buffer (Beyotime, Shanghai, China) containing 1 mM PMSF (Beyotime, Shanghai, China), protease inhibitor cocktail, (Beyotime, Shanghai, China) and phosphatase inhibitors (Beyotime, Shanghai, China). Cytosol and nucleus proteins were extracted using a nuclear and cytoplasmic protein extraction kit (Beyotime, Shanghai, China). The concentrations of protein were determined by the BCA Protein Assay Kit (Thermo Scientific, LOT UA276918, USA). Proteins were separated by 10% SDS-PAGE and transferred to nitrocellulose membranes (0.45 *μ*m) (Millipore, Ireland, LOT R1BB08547). The membranes were incubated with the following antibodies: *β*-catenin (1 : 1,000; CST 8480S), Lamin-b1 (1 : 1,000; CST 13435S), and GAPDH (1 : 1,000; CST 5174T). Subsequently, all membranes were incubated with horseradish peroxidase-conjugated secondary antibodies (CST 7074 S) for 2 hr at room temperature. The bands were detected using the Chemiluminescence imaging system (BG-gdsAUTO710Pro, Shanghai) with Pierce^TM^ ECL Western Blotting Substrate (Thermo, #32106) and quantified using the Image J software (National Institutes of Health, USA).

### 2.9. Immunofluorescence Staining

Cells were fixed with 4% paraformaldehyde (Biosharp, LOT 1810898) at room temperature and permeabilized with 0.1% Triton X-100 (Beyotime, Shanghai, China) for 15 min. Then, they were blocked with 5% normal goat serum for 60 min at room temperature and incubated with *β*-catenin (1 : 100; CST 8480 S) overnight at 4°C. Subsequently, they were incubated with the secondary antibody (1 : 750; Beyotime, Shanghai, China) for 1 hr at room temperature and stained with DAPI (Beyotime, Shanghai, China) for 5 min. Images were taken by Olympus microscope (Olympus IX73, Olympus, Japan).

### 2.10. Mice Femoral Fracture Models

This animal experiment was approved by the Institutional Animal Care and Use Committee of Guangzhou University of Chinese Medicine (Guangzhou, China). The C57BL/6 mice (12 weeks old, male, 25–30 g) were obtained from the Laboratory Animal Research Centre, Guangzhou University of Chinese Medicine (Guangzhou, China). The femoral fracture model was established according to previously validated protocols [25] and the size of incision was adjusted to 0.5 cm in mice. Thirty-six animals were randomly divided into three groups: control group; low-dose Aucubin group (0.5 mg/kg) and high-dose Aucubin group (1.0 mg/kg). Aucubin was suspended in isotonic saline, and the low-dose and high-dose groups were injected intramuscularly in the fracture sites with 0.5 mg/kg and 1.0 mg/kg every other day, respectively. The control group was injected intramuscularly with equal volumes of 0.9% normal saline at the same time points. All the mice were sacrificed at weeks 4 and 6. All femurs were collected for further analysis following X-ray radiography examination.

### 2.11. Micro-Computer Tomography (Micro-CT) Scanning

After the mice were sacrificed, their femurs were scanned using a micro-computer tomography (micro-CT) (Brucker, Skyscan1172, Belgium). The parameters of the micro-CT scanner were 5 *μ*m, 2,000 × 1,332 pixels. Measurements of femurs were based on 600 slices centered on the fracture site. The fracture callus and the adjacent bone were scanned using the same settings. The region of interest (ROI) was designated as 2.2 mm above and below the fracture plate in the present study. The 3D model was reconstructed and analyzed by the CT-VOX software (CtAN, Bruker, German). Subsequently, morphometric indices including total volume (TV), bone volume (BV), and bone volume/total volume (BV/TV) were calculated.

### 2.12. Bone Histomorphometry and IHC Staining

The fracture samples were fixed with 4% paraformaldehyde (Biosharp, LOT 1810898), and decalcified in 14% EDTA (solarbio, Beijing) for 7 days. Then, they were dehydrated and vitrified by dimethylbenzene. Following paraffin embedding, femurs were sectioned at 5 *μ*m and stained with Hematoxylin and eosin (H&E) and immunohistochemistry (IHC) using *β*-catenin (1 : 100; CST 8480S), osterix (OSX; 1 : 100; ab209484) and osteocalcin (OCN;1 : 100; ab93876) as primary antibodies. Sections were scanned with by digital section scanner (Pannoramic MIDI).

### 2.13. Establishment of the Stable H19-Knockdown MSCs

A lentiviral system was used to establish the H19 knockdown MSCs. Briefly, shH19 and the control shNC plasmids were, respectively, transferred into HEK293 cells with two packaging vectors pSPAX2 and pCMV-VSVg. The lentivirus was collected and infected MSCs. After screening with 2 *μ*g/mL puromycin for about 1 week, the H19 stably knockdown cells were generated.

### 2.14. Statistical Analysis

We used the IBM SPSS Statistics 24.0 to collect and analyze the data, and the GraphPad prism 8.0 was used to visualize the data. A two-tailed Student's *t*-test was used for statistical analysis between Aucubin and control groups. One-way ANOVA was performed to determine significant differences between intergroups analyses. At least, triplicates were taken in each experiment. The *P* value was set at a value of <0.05. Results are presented as means ± SD.

## 3. Results

### 3.1. Aucubin Had Nonobvious Effects on Cell Viability in BM-MSCs and RAW264.7 Cells

To investigate the effects of Aucubin on the differentiation of osteoblast and osteoclast, we first examined its effects on cell viability. Aucubin with various concentrations was added into BM-MSCs and Raw264.7 cells, and their viabilities were evaluated by CCK-8 assays. As shown in Figure S1, no obvious effects of Aucubin on cell viability were observed in BM-MSCs as well as RAW264.7 cells, suggesting Aucubin has low cytotoxicity.

### 3.2. Aucubin Promoted Osteoblast Differentiation *In Vitro*

**Figure 1:**
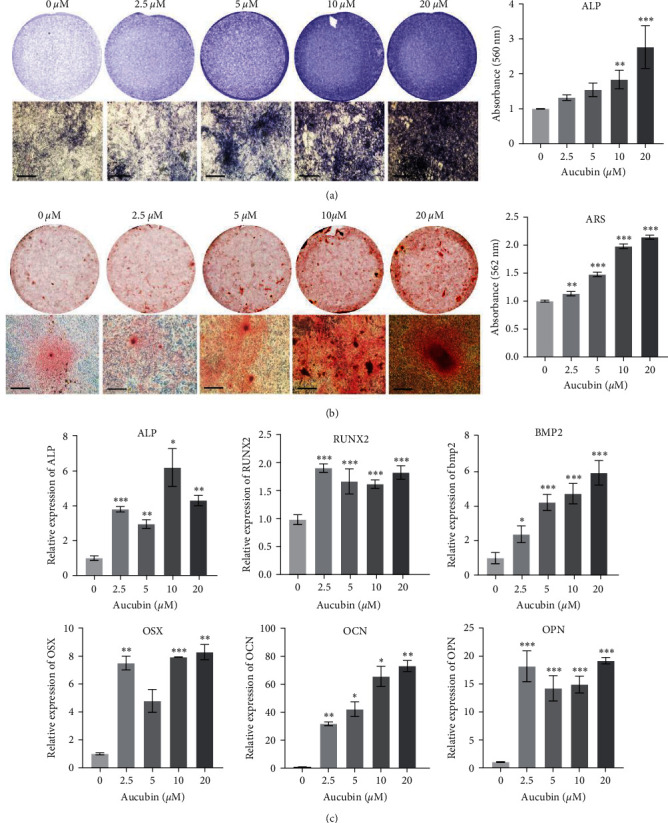
Aucubin promoted osteogenic differentiation of BM-MSCs. (a) Qualitative and quantitative examination of LP activity on day 7. (b) Qualitative and quantitative assays of ARS staining on day 21. Scale bar: 200 *μ*m. (c) The expression of osteogenic marker genes was measured on day 7. All experiments were repeated three times.  ^*∗*^*P*  < 0.05;  ^*∗∗*^*P*  < 0.01;  ^*∗∗∗*^*P*  < 0.001.

We next examined the effects of Aucubin on osteoblast differentiation. Under the osteogenic induction, BM-MSCs were treated with various concentrations of Aucubin. The ALP activity, an early marker of osteogenic differentiation, was assayed on day 7 and the results showed that it was enhanced by Aucubin in a dose-dependent manner, especially for 10 and 20 *μ*M Aucubin (Figure 1(a)). The further examination showed that the formation of calcium nodules was also promoted by Aucubin at day 21 (Figure 1(b)). We next examined the expression of osteogenic markers, including Runx2, OSX, BMP2, ALP, OCN, and OPN by qRT-PCR assays, and the results showed that all of them were significantly upregulated by Aucubin at day 7 (Figure 1(c)).

### 3.3. Aucubin Slightly Suppressed Osteoclast Differentiation *In Vitro*

On the other hand, the effects of Aucubin on osteoclast were also examined. With 40 *μ*M Aucubin treatment, RAW264.7 cells were induced to osteoclast differentiation, and the results demonstrated that the osteoclast differentiation was slightly suppressed using TRAP staining and F-actin staining examination (Figure S2). Furthermore, several crucial genes related to osteoclast differentiation such as MafB and RANK were examined and their expression was significantly decreased by Aucubin treatment (Figure S2). All these data suggested that Aucubin might slightly inhibit osteoclast differentiation.

### 3.4. Aucubin Induced H19 Upregulation in BM-MSCs

**Figure 2:**
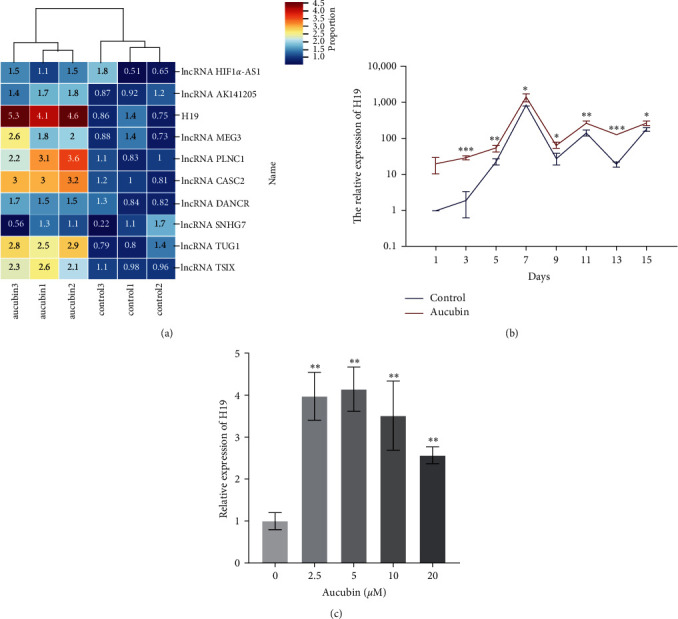
H19 was significantly upregulated by Aucubin in BM-MSCs. (a) Several lncRNAs related to osteogenesis were screened for examining their expression profiling in the MSC treated with Aucubin. (b) The H19 expression was further detected in the Aucubin-treated MSCs (0, 2.5, 5, 10, 20 *μ*M). (c) The H19 expression was examined during osteogenesis. *n* = 3;  ^*∗*^*P*  < 0.05;  ^*∗∗*^*P*  < 0.01;  ^*∗∗∗*^*P*  < 0.001.

lncRNAs have been considered as crucial regulators in cell differentiation. We justly speculated that lncRNAs may participate in Aucubin-mediated osteoblast differentiation. Several lncRNAs related to osteogenic differentiation were screened, and their expression profiling was detected. As shown in Figure 2(a), H19, lncRNA MEG3, lncRNA PLNC1, lncRNA CASC2, lncRNA TUG1, and lncRNA TSIX were upregulated by Aucubin treatment in BM-MSCs. Among them, H19 was the most changeable. This lncRNA was significantly upregulated in a concentration-dependent manner (Figure 2(b)). Furthermore, the expression pattern of H19 was examined during the osteoblast differentiation, and we found that it was obviously upregulated by Aucubin treatment (Figure 2(c)).

### 3.5. Aucubin Induced the Activation of the Wnt/*β*-Catenin Signaling

**Figure 3:**
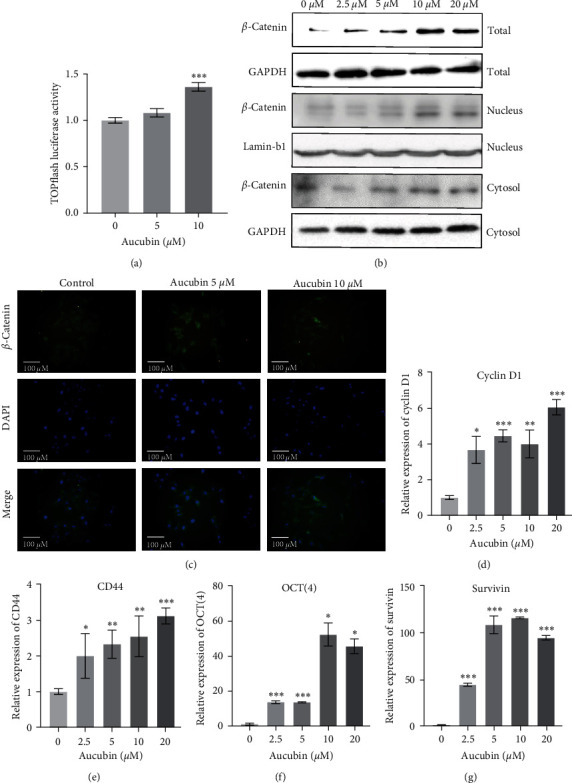
Aucubin stimulated the activation of Wnt/*β*-catenin signaling in osteogenic differentiation. (a) Luciferase activity was examined in the MSCs transfected with TOPFlash firefly luciferase reporter and TK-Renilla reporter with Aucubin treatment. (b) The expression levels of total, nuclear, and cytoplasmic *β*-catenin were detected by western blotting with Aucubin (0, 2.5, 5, 10, 20 *μ*M) treatment. (c) *β*-catenin expression was also detected by immunofluorescence staining. (d–g) The expression of several downstream genes of Wnt/*β*-catenin signaling was detected by qRT-PCR at day 7. All experiments were repeated three times.  ^*∗*^*P*  < 0.05;  ^*∗∗*^*P*  < 0.01;  ^*∗∗∗*^*P*  < 0.001.

Wnt/*β*-catenin signaling is a well-known transduction pathway and it plays critical role in bone formation [26]. H19 was reported to stimulate Wnt/*β*-catenin signaling and facilitated osteogenic differentiation in our previous study [27]. We therefore wondered whether this Wnt/*β*-catenin signaling participated in the Aucubin-induced osteoblast differentiation. As shown in Figure 3(a), the luciferase activity of TOPflash, a reporter of Wnt signaling, was examined and it was found that 10 *μ*M Aucubin significantly promoted this luciferase activity. Moreover, the expression of *β*-catenin, a key component of the Wnt signaling was significantly activated by Aucubin in BM-MSCs (Figure 3(b), Figure S3). The translocation and aggregation of *β*-catenin within the nucleus are tightly linked to the activation of Wnt/*β*-catenin signaling. We further examined the protein level of intranuclear *β*-catenin and intracytoplasmic *β*-catenin. Both the expression of intracytoplasmic and nuclear *β*-catenin was increased in a concentration-dependent manner (Figure 3(b), Figure S3). The immunofluorescence examination also confirmed that the expression of *β*-catenin was promoted with Aucubin treatment (Figure 3(c)). Furthermore, several downstream target genes of Wnt/*β*-catenin signaling such as cyclin D1, CD44, Oct3/4 and survivin were examined and the results showed that their expression was significantly upregulated by Aucubin (Figure 3(d)–3(g)). All these results suggested that Aucubin stimulated the canonical Wnt/*β*-catenin pathway *via* triggering its translocation from the cytoplasm to the nucleus.

### 3.6. Aucubin Accelerated Bone Fracture Healing *In Vivo*

**Figure 4:**
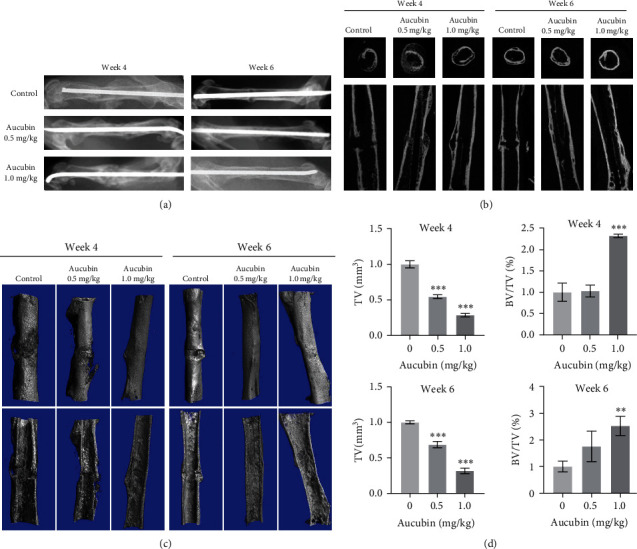
Aucubin facilitated bone fracture healing *in vivo*. The femoral fracture was created in C57BL/6J mice and subsequently treated with Aucubin for 4 or 6 weeks of low-dose (0.5 mg/kg), high-dose (1.0 mg/kg), or stroke-physiological saline solution. (a) Representative X-ray images were taken during the fracture healing processes at weeks 4 and 6. (b, c) Micro-CT examination of the femur fractured zone in Aucubin-treated groups. (d) The statistical diagram of TV and BV/TV at weeks 4 and 6. *n* = 3.  ^*∗∗*^*P*  < 0.01 and  ^*∗∗∗*^*P*  < 0.001.

**Figure 5:**
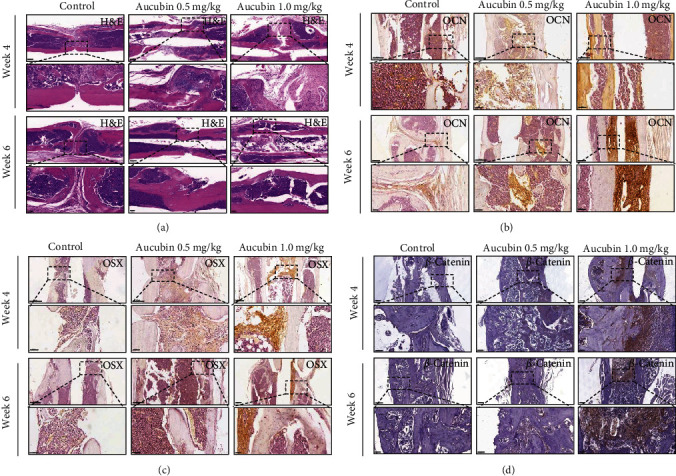
Aucubin faciliatted fracture healing by histological examination. (a) H&E staining assays for the fractured femur section at weeks 4 and 6. Immunohistofluorescence examination of OCN (b), OSX (c), and *β*-catenin (d) in the fractured femur section at weeks 4 and 6. Scale bars: 200 *μ*m (upper panel) and 50 *μ*m (lower panel, inset).

To evaluate the *in vivo* role of Aucubin, a femoral fracture model was established and Aucubin was locally injected into the fracture sites. The results of X-ray examinations showed a noticeably larger callus formation in the Aucubin-treated group compared with the control group at week 4 (Figure 4(a)). As time went on, the gap in the fracture sites nearly disappeared in the Aucubin group while it remained clearly in the control group at week 6, indicating that Aucubin facilitated the fracture healing process (Figure 4(a)). The further micro-CT scanning and the 3D reconstructed images demonstrated that thicker calluses were observed in the Aucubin-treated group at week 4, and the fracture healing were accelerated in treated groups at week 6 (Figures 4(b) and 4(c)). The data of tissue volume (TV) and bone volume (BV) were recorded, and quantitative analyses revealed that a significant increase in BV/TV in Aucubin-treated mice (Figure 4(d)). We further assessed the newly formed bone tissues by histological examination, and the results showed that bigger calluses were formed in the fracture sites and more active bone regeneration in Aucubin-treated groups (Figure 5(a)). By immunohistochemistry staining, the increased OCN and OSN expression was observed in Aucubin-treated group (Figures 5(b) and 5(c)), and *β*-catenin expression was also increased in treated group as well (Figure 5(d)).

### 3.7. Aucubin Promoted Osteoblast Differentiation *via* the H19/*β*-Catenin Regulatory Axis

**Figure 6:**
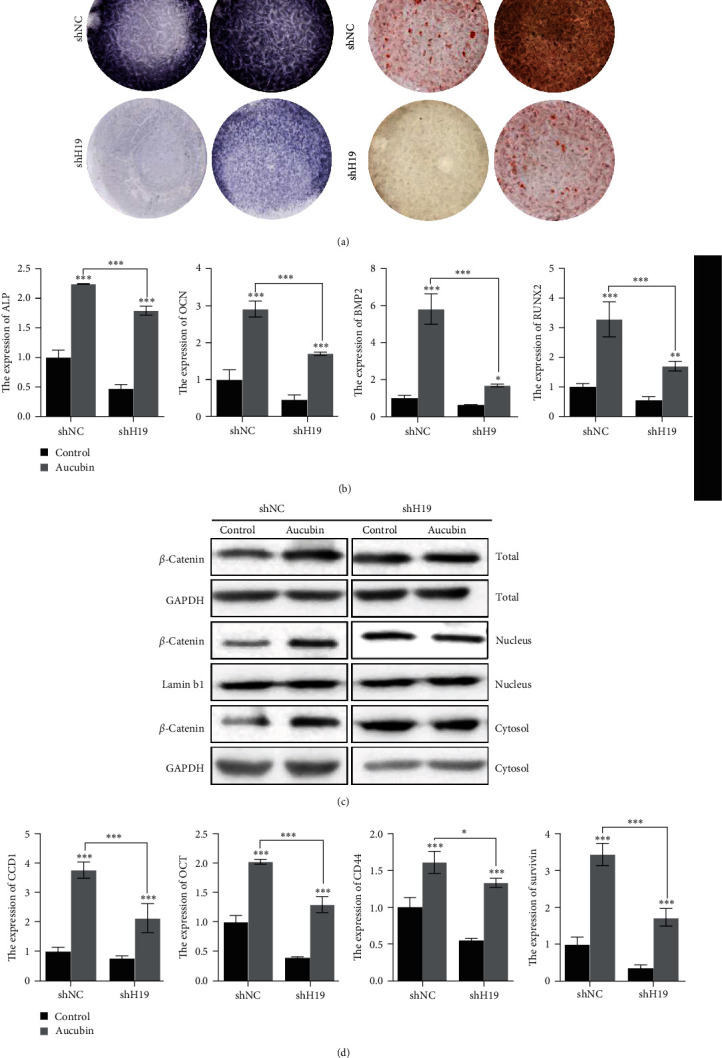
Aucubin promoted osteoblast differentiation *via* the H19-Wnt/*β*-catenin regulatory axis. (a) The ALP activity and ARS staining were examined in the Aucubin-treated shH19 MSCs. (b) The expression levels of osteogenic marker genes in the Aucubin-treated shH19 MSCs was examined. (c, d) The protein levels of total, nuclear, and cytoplasmic *β*-catenin were examined in the shH19 MSCs with Aucubin treatment. (d) The downstream genes of Wnt signaling were also detected in this Aucubin-treated shH19 MSCs. *n* = 3;  ^*∗*^*P*  < 0.05;  ^*∗∗*^*P*  < 0.01;  ^*∗∗∗*^*P*  < 0.001.

**Figure 7:**
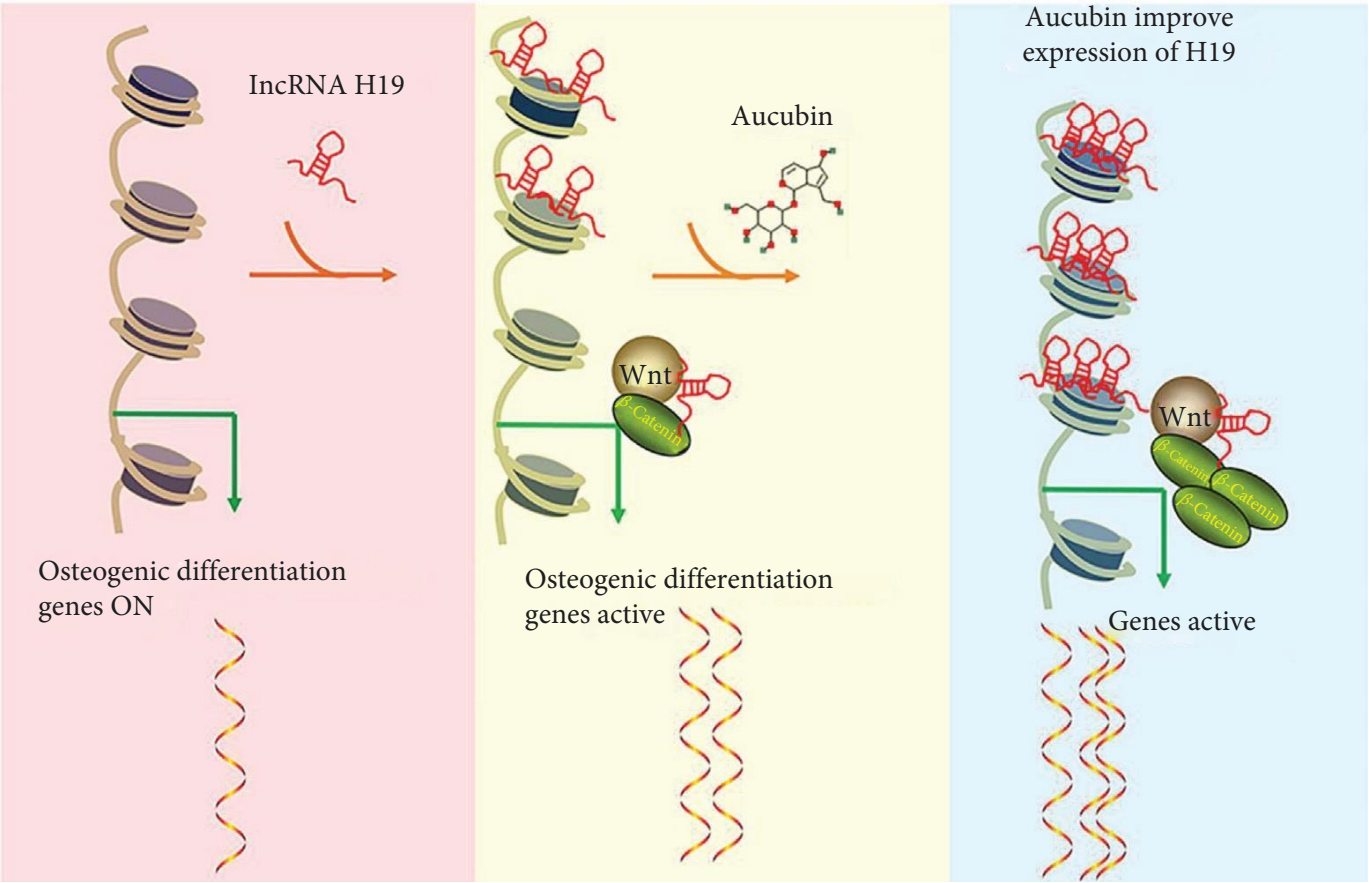
A schematic diagram of the mechanism illustrated in this study. Aucubin promoted osteogenic differentiation and facilitated bone formation through the lncRNA-H19 driven Wnt/*β*-catenin signaling regulatory axis, in which H19 was obviously upregulated by Aucubin, and stimulated the activation of Wnt/*β*-catenin signaling, eventually leading to facilitating osteogenic differentiation.

To further identify the role of H19 in Aucubin-mediated osteogenic differentiation, two H19 knockdown MSCs were generated and qRT-PCR examinations were used for evaluating the silenced effects. It was found that H19 expression was obviously suppressed in the two H19 silenced MSCs (Figure S4). With Aucubin treatment, the H19-knockdown cells were induced to osteoblast differentiation, and we observed that H19 knockdown partially attenuated the effects of Aucubin on osteogenic differentiation by the examination of ALP activity, ARS staining, and expression of osteogenic genes (Figures 6(a)and 6(b)). It was also found that Aucubin-elevated *β*-catenin expression was partially reversed by H19 knockdown (Figure 6(c), Figure S5), and the increased downstream targets were partially alleviated as well (Figure 6(d)). Based on all the results, a schematic overview showed as follows: Aucubin significantly promoted H19 expression which served as an activator of Wnt signaling, and stimulated the activation of Wnt/*β*-catenin signaling, eventually leading to facilitating osteogenic differentiation and bone formation (Figure 7).

## 4. Discussion

Fractures are common orthopaedic diseases, which bring heavy pain to patients and enormous burdens to social health care systems [2–4]. Aucubin, a kind of natural flavone derived from cortex *Eucommiae*, possesses extensive biological effects such as anti-inflammatory neuroprotective and osteoprotective properties [8, 9]. Further studies have demonstrated that Aucubin plays a protective role in bone metabolism *via* various mechanisms [10–12]. For instance, Aucubin promoted autophagy *via* AMPK activation and thus prevented steroid-induced osteoblast apoptosis [11]; it also inhibited osteoclast differentiation *via* the nuclear factor erythroid 2-related factor 2-mediated antioxidation pathway and thus alleviated osteoporosis [27]. The beneficial impacts of Aucubin on osteogenic differentiation are well established. For instance, Aucubin stimulated the activations of Akt and MAPKs pathways to promote osteoblast differentiation [9], and it also activated Nrf2/HO-1 signaling to exert antiosteoporotic effects [10]. In the present study, Aucubin was found to promote osteogenesis *in vitro* and accelerate bone formation *in vivo*, which was consistent with already published data. However, our data also showed that lncRNA H19 was obviously upregulated by Aucubin in MSCs and thus induced the activation of Wnt/*β*-catenin signaling, which provides a novel mechanism underlying Aucubin-mediated osteogenic differentiation.

It is well-established that Wnt/*β*-catenin signaling plays a pivotal role in the differentiation of cells and the formation of the skeletal matrix during skeletal development [27–29]. In the present study, our results showed that Aucubin increased the expression of *β*-catenin and downstream genes of Wnt signaling, indicating that it may activate the Wnt/*β*-catenin pathway. However, the detailed mechanism underlying Aucubin-mediated Wnt/*β*-catenin signaling remained obscure. As a new member of epigenetic regulators, many lncRNAs have been reported to regulate osteogenic differentiation through Wnt/*β*-catenin cascades [30–32]. Several lncRNAs related to osteogenic differentiation were screened, and their expression was detected in the present study. Our results showed that H19 was the most upregulated by Aucubin in MSCs. Previous studies have shown that H19 was upregulated in osteogenic differentiation and its elevation promoted bone formation [22, 23, 33], which was consistent with our results. Simultaneously, H19 also inhibited the adipogenic differentiation of BMSCs [34]. Therefore, H19 may be a potential therapeutic target for bone fracture. H19 was reported to directly target P53 and sponges miR-138 to stimulate osteogenesis [35, 36]. It also reported that H19 served as a competing miRNA for miR-22, miR-141, and miR-675 to mediate Wnt/*β*-catenin signaling. The activation of the H19-Wnt/signaling pathway is critical for bone formation and regeneration; and disruption of this pathway could inhibit bone repair in the early stage of fracture healing [23]. Our results demonstrated that H19 knockdown partially attenuated the Aucubin-promoted osteoblast differentiation as well as the activation of Wnt/*β*-catenin signaling. All these data indicated that Aucubin promoted osteogenesis through the H19/Wnt/*β*-catenin signaling axis. Interestingly, Zhu et al. [12] reported that BMP2/Smads/RunX2 signaling was involved in the Aucubin-mediated osteogenesis, suggesting multitargets of Aucubin in osteoblasts. This crosstalk between BMP2 and Wnt signaling has been confirmed in myoblasts and neuronal differentiation [37, 38]. Combined with our results and abovementioned publication, the potential interplay between H19/Wnt/*β*-catenin signaling and BMP2/Smads/RunX2 signaling may be a synergistic mechanism of Aucubin in osteogenic differentiation.

To further explore the effect of Aucubin *in vivo*, an established femoral fracture model in mice was applied [25]. Twelve-week-old (3 months) mice were chosen to make this fracture model using an open femoral osteotomy after intramedullary stabilization. Aucubin was locally injected into fracture sites to evaluate the healing process. The results indicated that Aucubin promoted the new bone formation and accelerated fracture healing based on the X-ray examination, micro-CT scanning, HE, and IHC staining. Mechanically, we observed more positive staining of *β*-catenin, OCN, and OSX in the Aucubin-treated group in the fracture zones. Considering that both osteocalcin (OCN) and osterix (OSX) could promote bone formation [39, 40], it could be found that Aucubin promoted bone formation by enhancing both osteocalcin (OCN) and osterix (OSX) expressions, indicating that Aucubin may be a promising compound to enhance bone formation and accelerate the repair of the fracture. According to the obtained results, we concluded that Aucubin could promote osteogenesis and accelerate bone fracture healing through stimulating the H19/Wnt signaling pathway.

In summary, our results indicated that Aucubin stimulated osteogenic differentiation of BM-MSCs *in vitro* and accelerated fracture healing *in vivo*. lncRNA H19 was upregulated by Aucubin, and then stimulated the Wnt/*β*-catenin signaling in osteogenic differentiation. Therefore, our findings demonstrated that Aucubin promoted bone formation through the H19-Wnt/*β*-catenin regulatory axis, suggesting that Aucubin may be a potential agonist to develop the bone-protective therapeutic strategy for clinical practice.

## Figures and Tables

**Table 1:** The primer sequences of osteoblast-related gene.

Gene name	Primer sequences (5′−3′)
mALP-F mALP-R mOPN-F mOPN-R mOsterix-F mOsterix-R mRUNX2-F mRUNX2-R mBMP-2-F mBMP-2-R mOCN-F mOCN-R hH19-F hH19-R TRAP-F TRAP-R mRANK-F mRANK-R mCatk-F mCatk-R mMafB-F mMafB-R	GCCCTCTCCAAGACATATA CCATGATCACGTCGATATCC TCACCATTCGGATGAGTCTG ACTTGTGGCTCTGATGTTCC GTGAATTCACCTTTCAGCCCCAAAACC TGGGATCCCAGCTGTGAATGGGCTTCTT GACTGTGGTTACCGTCATGGC ACTTGGTTTTTCATAACAGCGGA AGTTCTGTCCCCAGTGACGAGTTT GTACAACATGGAGATTGCGCTGAG CCTCAGTCCCCAGCCCAGATCC CAGGGCAGAGAGAGAGGACAGG ATCGGTGCCTCAGCGTTCGG CTGTCCTCGCCGTCACACCG TCATGGGTGGTGCTGCT GCCCACAGCCACAAATCT CCAGGAGAGGCATTATGAGCA ACTGTCGGAGGTAGGAGTGC AATACGTGCAGCAGAACGGAGGC CTCGTTCCCCACAGGAATCTCTCTGTAC GAGCAGGTGTGACTCACGAT TGGCTAGTGGGTAGCTGTTG

## Data Availability

The original data presented in the study are included in the article/supplementary material, further inquiries can be directed to the corresponding authors.

## Supplementary Materials

Supplementary Materials Figure S1: cell viabilities of BM-MSCs and RAW264.7 were examined with Aucubin treatment. Figure S2: Aucubin slightly suppressed osteoclast differentiation. Figure S3: the quantitative assays of *β*-catenin expression in the Aucubin-treated MSCs. Figure S4: H19 was significantly suppressed by two shH19 plasmids infected MSCs. Figure S5: the quantitative examination of *β*-catenin in Aucubin-treated shH19 MSCs.
