# Supplementary material for: Aucubin Promotes Osteogenic Differentiation and Facilitates Bone Formation through the lncRNA-H19 Driven Wnt/β-Catenin Signaling Regulatory Axis
Source: Stem Cells Int. 2024 Apr 9;2024:5388064. doi: 10.1155/2024/5388064 (PMC11022505; doi:10.1155/2024/5388064)
Supplement: Supplementary Materials — Figure S1: cell viabilities of BM-MSCs and RAW264.7 were examined with Aucubin treatment. Figure S2: Aucubin slightly suppressed osteoclast differentiation. Figure S3: the quantitative assays of β-catenin expression in the Aucubin-treated MSCs. Figure S4: H19 was significantly suppressed by two shH19 plasmids infected MSCs. Figure S5: the quantitative examination of β-catenin in Aucubin-treated shH19 MSCs. [file 5388064.f1.docx]

Supplementary Figures

**Supplementary Figure S1.** **Cell viabilities of BM-MSCs and RAW264.7 were examined with Aucubin treatment.** (A), BM-MSCs were treated with various concentrations of Aucubin (0, 2.5, 5, 10, 20μM), and cell viabilities were examined by CCK8 kit. (B), RAW264.7 cells were treated with various concentrations of Aucubin (0, 10, 20, 40, 80, 160μM), and cell viabilities were examined. n=3.


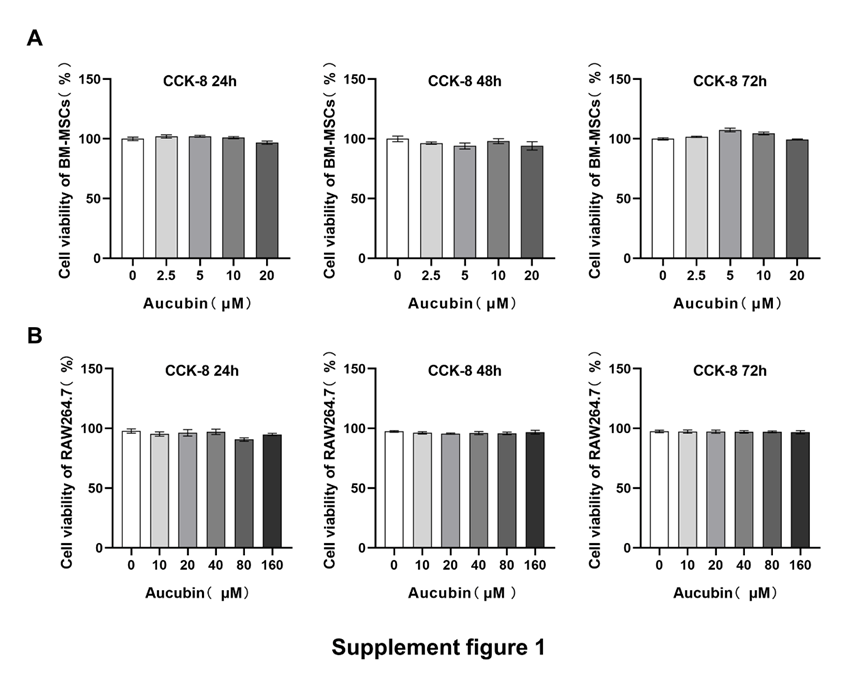


**Supplementary Figure S2.** **Aucubin slightly suppressed osteoclast differentiation*.*** (A-B), TRAP staining was performed in the Aucubin-treated Raw264.7 cells (A) and the numbers of positive cells (≥3 nuclei) were statistically recorded (B). **(C)**, The staining of rhodamine-phalloidin for F-actin ring in the treated cells. **(D)**, The expression of several markers related to osteoclast differentiation was examined by qRT-PCR assays on day 5. All experiments were repeated three times. n=3; *, P< 0.05; **, P< 0.01; ***, P< 0.001.


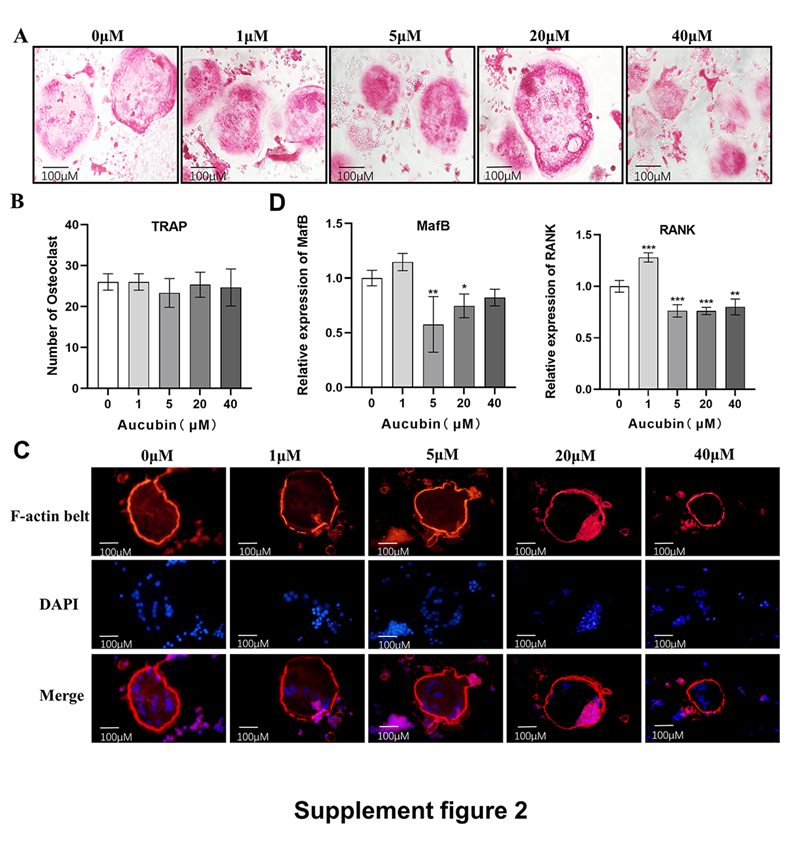


**Supplementary Figure S3. The quantitative assays of β-catenin expression in the Aucubin-treated MSCs.** The expression levels of total, nuclear and cytoplasmic β-catenin were examed by Western blotting with various concentrations of Aucubin(0, 2.5, 5, 10, 20μM) treatment and the quantitative assays were performed using ImageJ. All experiments were repeated three times. n=3; **, P< 0.01; ***, P< 0.001.


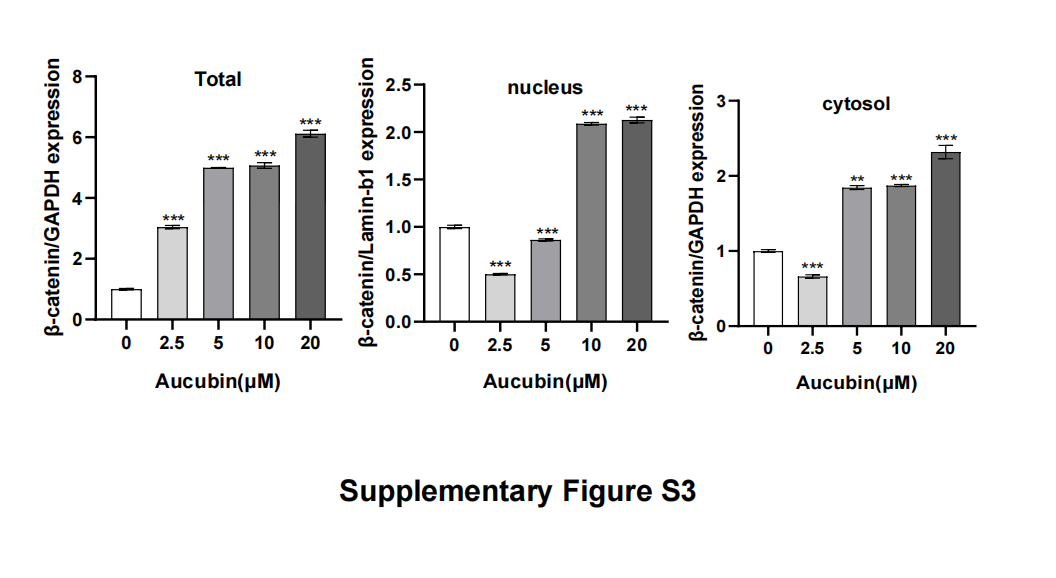


**Supplementary Figure S4. H19 was significantly suppressed by two shH19 plasmids infected MSCs.** H19 knockdown stable cell lines were constructed using lentiviral system. Subsequently, the knockdown efficiency was detected by RT-QPCR. n=3; **, P< 0.01.


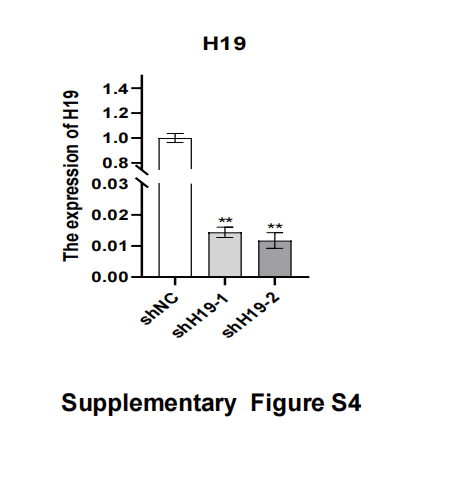


**Supplementary Figure S5.** **The quantitative examination of β-catenin in Aucubin-treated shH19 MSCs.** The expression levels of total, nuclear and cytoplasmic β-catenin were detected by Western Blotting. Subsequently, quantitative assay were conducted using ImageJ. (A), Total β-catenin expression. (B), Nuclear β-catenin expression. (C), Cytoplasmic β-catenin expression. n=3; ***, P< 0.001.


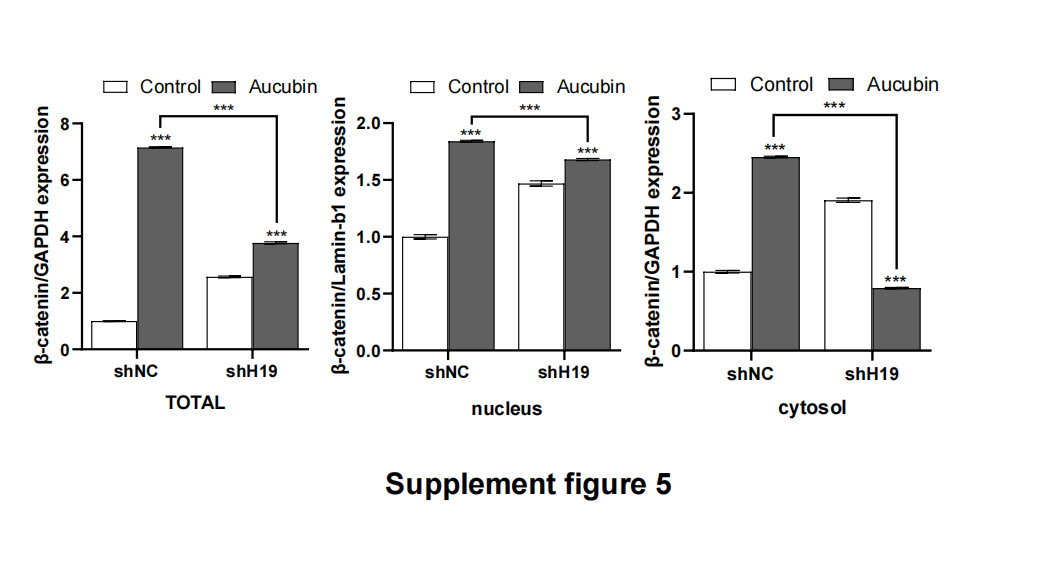


**Conflict of Interest Statement**

All authors declared no potential conflicts of interest.
